# A screen-based simulation training program to improve palliative care of people with advanced dementia living in residential aged care facilities and reduce hospital transfers: study protocol for the IMproving Palliative care Education and Training Using Simulation in Dementia (IMPETUS-D) cluster randomised controlled trial

**DOI:** 10.1186/s12904-019-0474-x

**Published:** 2019-10-23

**Authors:** Joanne Tropea, Christina E. Johnson, Debra Nestel, Sanjoy K. Paul, Caroline A. Brand, Anastasia F. Hutchinson, Ross Bicknell, Wen Kwang Lim

**Affiliations:** 1 Melbourne EpiCentre, Royal Melbourne Hospital, University of Melbourne and Melbourne Health, 7 East Main building, 300 Grattan Street, Parkville, VIC 3050 Australia; 2 Monash Doctors Education, Monash Health Faculty of Medicine, Nursing and Health Sciences, Monash University, Monash Medical Centre, 246 Clayton Rd, Clayton, VIC 3168 Australia; 3 Monash Institute for Health & Clinical Education, Monash University, 27 Rainforest Walk Wellington Road, Clayton, VIC 3800 Australia; 4 School of Nursing and Midwifery, Deakin University, Locked Bag 20000, Geelong, VIC 3220 Australia; 5 Department of Medicine and Aged Care, Melbourne Health and Department of Medicine - Royal Melbourne Hospital, University of Melbourne, 300 Grattan Street, Parkville, VIC 3050 Australia

**Keywords:** Dementia, Cluster RCT, Palliative care, End-of-life care, Simulation training, Care worker education, Nursing education, Long-term care homes; nursing homes, Quality of care, Process evaluation

## Abstract

**Background:**

Many people with advanced dementia live in residential aged care homes. Care home staff need the knowledge and skills to provide high-quality end-of-life (EOL) dementia care. However, several studies have found EOL dementia care to be suboptimal, and care staff have reported they would benefit from training in palliative care and dementia. Simulation offers an immersive learning environment and has been shown to improve learners’ knowledge and skills. However, there is little research on simulation training for residential care staff.

This article presents the development and evaluation protocol of IMproving Palliative care Education and Training Using Simulation in Dementia (IMPETUS-D) - a screen-based simulation training program on palliative dementia care, targeted at residential care staff. IMPETUS-D aims to improve the quality of palliative care provided to people living with dementia in residential care homes, including avoiding unnecessary transfers to hospital.

**Methods:**

A cluster RCT will assess the effect of IMPETUS-D. Twenty-four care homes (clusters) in three Australian cities will be randomised to receive either the IMPETUS-D intervention or usual training opportunities (control). The primary outcome is to reduce transfers to hospital and deaths in hospital by 20% over 6-months in the intervention compared to the control group. Secondary outcomes include uptake of goals of care plans over 6 and 12 months, change in staff knowledge and attitudes towards palliative dementia care over 6 months, change in transfers to hospital and deaths in hospital over 12 months. For the primary analysis logistic regression models will be used with standard errors weighted by the cluster effects. A mixed methods process evaluation will be conducted alongside the cluster RCT to assess the mechanisms of impact, the implementation processes and contextual factors that may influence the delivery and effects of the intervention.

**Discussion:**

In Australia, the need for high-quality advanced dementia care delivered in residential aged care is growing. This study will assess the effect of IMPETUS-D a new simulation-based training program on dementia palliative and EOL care. This large multisite trial will provide robust evidence about the impact of the intervention. If successful, it will be distributed to the broader residential care sector.

**Trial registration:**

ANZCTR, ACTRN12618002012257. Registered 14 December 2018.

## Background

Dementia is a progressive, terminal neurological disease. As dementia progresses, cognition and physical function declines and care needs increase, often resulting in admission to residential care. In Australia, residential care is a key provider of palliative and end-of-life (EOL) care to people with advanced dementia, with over 50% of people living in residential care having a confirmed diagnosis of dementia [1]. This number is likely to be an under-estimate due to the under-diagnosis of dementia.

Features of high-quality palliative care for people with dementia includes: a person-centred approach, clear communication, shared decision making including setting care goals and advance care planning, optimal treatment of symptoms, continuity of care, psychosocial and spiritual support, family care and involvement, societal and ethical issues, and prognostication and timely recognition of dying [2]. However, several studies have shown people with advanced dementia living in residential care often receive suboptimal palliative care - advance care planning is not adequately addressed, unnecessary transfers to hospital occur, uncomfortable interventions are common, and distressing symptoms are not always adequately recognised or managed [3, 4].

Studies have also reported health care workers who provide care to people with dementia lack confidence in their skills, and recommend educational strategies to improve the quality of palliative and EOL dementia care [5, 6]. This was highlighted in a survey conducted by Alzheimer’s Australia, in which 90% of care professionals indicated that additional training on palliative care and dementia would be beneficial [4].

Although those providing palliative care to people with dementia feel they would benefit from additional training, there is limited evidence to support use of palliative care training. Previous studies have shown either no impact on clinical outcomes or, have been of low methodological quality with high risk of bias due to study design [7, 8]. This was highlighted in a narrative review by Raymond et al. (2014) where dementia-specific palliative care training failed to show benefits for people with dementia [8].

The palliative care training and education interventions assessed in previous studies used train-the-trainer, and workshops with predominantly didactic presentations. Simulation is an innovative training concept in the residential care workforce. Simulation-based training offers an immersive learning environment that facilitates deliberate practice. There are many types of simulation modalities with technological developments fostering the uptake of screen-based approaches. That is, simulations that are viewed on computers, tablets or smartphones, offering features such as audio-visual sharing and capture of information, repetitive practice and feedback for both individual and group use, that are available when and where they are needed assuming internet and other infrastructure is accessible. A screen-based approach offers a way to upskill the busy and often time poor residential aged care workforce as it can be accessed at their convenience.

In settings other than residential care, simulation-based palliative care training has been shown to improve learners’ knowledge and skills [9–11], but very few studies have assessed the impact of these training interventions on patient or clinical outcomes. Further, to the best of our knowledge, high quality simulation-based research including screen-based simulation in the residential care setting is limited [12].

In this study the effect of a new training program IMproving Palliative care Education and Training Using Simulation in Dementia (IMPETUS-D) will be assessed. IMPETUS-D is a screen-based simulation training program designed to improve care for people with advanced dementia living in residential care. The targeted learners will be nursing and care attendant staff in residential care settings.

This study will use a cluster randomised controlled trial (RCT) design, in parallel with a process evaluation using mixed-methods research. The primary hypothesis is that residential care facilities that participate in the IMPETUS-D training program will reduce the proportion of transfers to hospital and/or deaths in hospital among residents with dementia by 20% over 6 months, compared with the control group.

## Methods/design

### Study design

This is a multi-centre, facility-level cluster RCT, with parallel mixed methods process evaluation. Residential aged care facilities (RACFs) will be randomly allocated to the intervention (IMPETUS-D program) or the control group (usual training).

### Study setting

The study will be conducted in 24 RACFs (12 intervention and 12 controls) of one Australian private aged care provider, across three Australian cities: Sydney, Melbourne and Adelaide.

### Recruitment of RACFs (clusters)

All RACFs that participate are part of a single Australian private aged care provider. The provider is a collaborative partner in the study. To be eligible for inclusion in the study RACFs needs to have at least 20 people with dementia permanently living in the care home and requiring high level care. The residential care homes that meet the inclusion criteria will be recruited into the study for randomisation and group allocation.

### Randomisation and blinding

Individual RACFs (clusters) in the trial will be randomised to training or control group, with staff at the RACF receiving either the intervention training or the control group. The allocation sequence will be generated by an independent statistician using a computer-generated allocation sequence. The randomisation will use blocks of 4 stratified by city location (Sydney, Melbourne or Adelaide) and facility size (small or large, based on the total number of residents). Small RACFs are defined as having 70 or less residents and large RACFs defined as having greater than 70 residents living in the care home. The randomisation list will be stored and maintained in a secure location by a statistician who is independent from trial recruitment or training so as to preserve blinding.

### Intervention

#### Development of the intervention

The intervention comprises 11 modules covering key aspects of high-quality palliative care for people with dementia towards the EOL. These topics were judged to be the most relevant for nursing and care staff, informed by multiple sources including, staff working in residential care, published literature, and the advice of the IMPETUS-D Project Working and Advisory Group which included palliative care, dementia and aged care experts. Experts in simulation education and health profession education developed the modules in accordance with principles of high quality online education [13, 14]. The aim is to increase staff motivation, competence and confidence into offering high quality care using interactive and simulation-based learning.

The modules can be used on mobile technologies as this format offers users greater convenience for access. They allow self-paced learning, easily re-entering the module at users’ convenience, for example if they run out of time or if want to review a specific video a few weeks later. The modules take into account variable education and literacy levels, and that English may not be the primary language of some care home staff.

The module content reflects common scenarios in residential care that raise opportunities or dilemmas for staff in providing high quality palliative care (known as ‘trigger narratives). Examples include outlining the features of useful and practical GOC plans or addressing myths around perceived barriers to providing adequate pain relief. The modules were designed to give the locus of control to users. They were provided with relevant knowledge and these illustrated and then applied to the narratives. The narratives also served as prompts for learner application and are in text, still image with text or video. In most modules, the central case scenario involves a brief video sequence of 1–2 min. This is accompanied by additional still images and text guiding staff through questions or how to competently accomplish key tasks, along with succinct relevant information, to encourage active learning.

Although each module was designed to stand alone, the story line of the trigger narratives has been threaded across modules. In addition, deliberate practice is encouraged by having different but somewhat overlapping tasks in different modules. An innovative feature, designed to enhance the ability and confidence of staff to communicate well with families, includes rehearsing their response to a question posed by a family member in the module scenario. They will be invited to record it on their phone, listen to it and reflect, then revise it until they feel satisfied with it and then listen to a good example response in the module. Staff will be invited to upload their own recordings and add comments, so a variety of anonymous authentic responses can be selected and made available on a users’ discussion board to provide additional education resources and encourage collaborative learning.

Module topics include the natural progression of dementia and how to recognise when deterioration is due to dementia versus delirium, recognising when a person with dementia is approaching the EOL, goals of care (GOC) plans and discussions, symptom management including management of pain, breathlessness, not eating or drinking, and terminal restlessness, communicating with residents and their families, and staff wellbeing when a resident dies. Further details on the modules can be found in Table 1.

**Table 1:** The overview of content and learning outcomes for each of the IMPETUS-D modules

Module title	Overview of content and targeted learners	When you’ve finished this module, you’ll be able to do 3 things:
1. Nancy just seems to be going downhill, for no reason …	This module describes the natural progression of dementia and how to recognise when deterioration is due to dementia versus delirium. The module introduces Nancy Thompson a resident with dementia, and in two video-based scenarios Nancy declines (i) due to the progression of dementia and (ii) due to an acute delirium. Targeted learners: PCAs	• Know more about the natural progression of advanced dementia • Recognise the difference between deterioration due to dementia or due to delirium • Document what you’ve noticed and pass this information to a nurse
2. Nancy, Bob and Ted: Getting care ‘just right for you’	This module focuses on caring for a person with advanced dementia when they are approaching the end of their life. It includes regular discussions with the family so everyone is on the same page, and focusing care on maximising wellbeing, comfort and dignity, and minimising distress. The module includes audio recordings of PCAs and family members talking about 3 residents who received suboptimal care, and asks learners to write down their thoughts on what could have been done differently. Targeted learners: PCAs	• Recognise when a person with advanced dementia is approaching the end of their life • Know what needs to be done at this stage • Know how to make valuable contributions to the care plan
3. “Should Antonio stay or should he go?”	This module discusses the reasons why people with advanced dementia are typically best cared for in the care home, and describes the challenges people with advanced dementia face in the busy hospital environment. The module introduces Antonio Conti a resident with dementia and his wife Bianca. It includes a video-based scenario where Bianca confronts a PCA when Antonio becomes sick, as she is very worried that Antonio may need to go into hospital, as last time he went to hospital, things did not go well. Targeted learners: PCAs	• Know that when a resident with advanced dementia gets sick, usually staying in the care home is the best place for them • Know the challenges for a resident with advanced dementia if they go to hospital
4. What would they choose?	This module gives an introduction to Goals of Care (GOC) plans, and highlights that finding out what’s important to the person is vital to providing best care. It follows the development of a GOC plan including a GOC discussion between Antonio, his family, the care manager and general practitioner. This module includes audio-recordings of a quality GOC discussion with Antonio’s GP and family; and audio-recordings from care home staff giving their perspective on GOC discussions. Targeted learners: nurses and care managers	• Describe a GOC plan • Identify the benefits of a GOC plan for a person with advanced dementia • Know how to discover and value what the resident wants or would have wanted if their advanced dementia had not impaired their decision making capacity
5. Goals of care plans – making them useful	This module describes what is needed to be a high quality and best practice Goals of care (GOC) plan. It provides examples of how to best use GOC plans for residents with dementia, and describes situations when a person with advanced dementia needs to go to hospital. In this module a video-based scenario involving Antonio Conti demonstrates there are exceptions that require transfer to hospital. Targeted learners: nurses and care managers	• Describe the features of a quality GOC plan • Recognise the importance of updating GOC plans regularly and that these plans be informed by discussions with the resident and their family • Identify the exceptional circumstances when residents with advanced dementia and nearing the end of their life, may benefit from going to hospital (e.g. fractured hip)
6. “At the end, I just don’t want any pain.”	This module discusses pain and the challenges in managing pain in people with advanced dementia. It highlights the importance of how to recognise it, document it, and act on it including speaking up for people with dementia who are in pain. In addition, it describes some of the myths and truths about opioid use. This module introduces Mrs. Keya Basu a resident with advanced dementia. It includes a video-based scenario where Mrs. Basu’s daughter Priya can hear her mother crying out in pain while a wound dressing is being changed. Targeted learners: PCAs	• Effectively assess and document pain in a person with advanced dementia • Speak up in support of a resident to get the pain relief they need • Know the myths and truths about opioid use
7. “My mother won’t eat or drink.”	This module focuses on recognising the dying phase, including reduced eating and drinking, and helping families understand that reduced eating and drinking at the end of life is normal. This module includes a video-based scenario where Mrs. Basu’s daughter Priya tells a PCA she is concerned her mother is not eating or drinking, and questions the need for a drip or feeding tube. Targeted learners: PCAs	• Know that reduced eating and drinking is normal at the end of life • Explain these end of life changes (e.g. reduced eating and drinking) to family members • Describe how to give good mouth care
8. The last few days were so precious	This module has 2 parts. The first discusses focussing on what is most important to the person when they are dying and ways to keep a person as comfortable as possible. The second part describes terminal restlessness a common end of life symptom, and how care staff can recognise and manage it. This module includes a scenario where Mrs. Basu’s daughter Priya is talking to a PCA about her mother being restless. Targeted learners: PCAs	• Offer ideas to families about making the most of the time left when a person is dying • Encourage family to participate in optimising care • Recognise and manage restlessness when a person is dying
9. “What if Mrs. Basu dies on my shift?”	This module focuses on changes in breathing at end of life, what to expect as death approaches and ways to support the family. It also discusses the concerns care staff may have as a resident approaches death and includes reflection of learners’ real experiences. This module includes a scenario with a PCA expressing concerns about “what if Mrs. Basu dies on my shift?” Targeted learners: PCAs	• Know what to expect as death approaches, including common breathing changes • List what needs to be done following death • Describe the importance of grieving a resident’s death and celebrating their life
10. “I didn’t know what to say …” - Communicating when a resident is nearing the end of their life	This module raises the importance of communicating openly, honestly and frequently with residents and their family members. Strategies for communication are offered for difficult conversations. Three video-based scenarios of challenging situations involving Antonio Conti’s family are presented. Targeted learners: all care staff	• Describe effective strategies for communicating with a resident with advanced dementia at the end of their life. • Describe effective strategies for communicating with key individuals (family members) about a resident with advanced dementia at the end of their life. • Use words and phrases that are sensitive and helpful in the period around the end of life.
11. “It can be so emotional … it can really take you down” - Managing your own feelings and wellbeing when a resident dies	This module shifts the focus of caring from residents to care providers. It encourages carers to consider their own feelings about death and dying and how they manage these feelings when a resident is at the end of their life and then dies. It offers strategies to manage emotional wellbeing and ways to contribute a compassionate culture in their workplace. The module includes audio recordings of PCAs talking about some real experiences, and encourages learners to role play a conversation that acknowledges feelings. Targeted learners: PCAs	• Describe your feelings when a resident is at the end of their life and why it is important to be aware of them • Develop strategies to manage your emotional wellbeing when a resident is at the end of their life • State ways to contribute to building a compassionate culture in your workplace

*Abbreviations*: *GOC* goals of care, *PCAs* personal care attendants/assistants

Each module has been designed to take approximately 15-30 min, and participants can access the training program at their discretion throughout the study timeframe. There will be no limit on the number of times staff access the training program. The training program can be accessed on a desktop computer, laptop, tablet or smartphone.

Modules 1–3, 6–9 and 11 have been designed for personal carers as the targeted learners; and Modules 4 and 5 have been designed for nurses as the targeted learners. Module 10 has been designed for all care home staff.

Personal carers (or PCAs) will be expected to complete at least 5 of the modules – Modules 1–3 and 10, and one symptom management module (Modules 6–9). The remaining modules on goals of care discussions, goals of care plans, and staff well-being will be recommended for them to complete. Nurses will be expected to complete the two goals of care modules and the communication module. All other modules will be recommended for nurses to complete. The aim is for 80% of staff to complete the ‘expected to complete’ modules.

The modules will be published in HTML5 (scorm 1.2) located on a learning management system (LMS) hosted by Kontent Labs propriety system [15]. The amount of usage (hits and time spent) will be collected via the LMS.

#### Implementation of the intervention

The implementation of IMPETUS-D has been guided by the Consolidated Framework for Implementation Research (CFIR) [16], the UK Medical Research Council process evaluation framework [17], and the Expert Recommendations for Implementing Change [18]. Due to the complexity of this large multi-site study, an implementation plan has been developed to increase the likelihood of successful implementation of the IMPETUS-D program. See Additional file 1 for outline of the implementation strategies that will be conducted as part of the study.

Three local Project Coordinators will be appointed, one in each city, to assist in the day to day running of the project including recruitment of RACF staff and implementation and data collection activities throughout the project timeline. The Project Coordinators will be nurses with experience working in residential care. They will receive training on the project data collection methods and implementation activities to ensure a standardised approach.

The IMPETUS-D program will be implemented over a 2-month training period during which time we anticipate most targeted staff at the intervention sites will complete the training program. During the training period Project Coordinators will actively engage with staff to promote and encourage their participation in the program, including sending weekly reminder texts and emails and attending staff meetings in-person where uptake is low. The 2-month training period will be followed by 12 months of prospective data collection of primary and secondary outcomes.

**Fig. 1:**
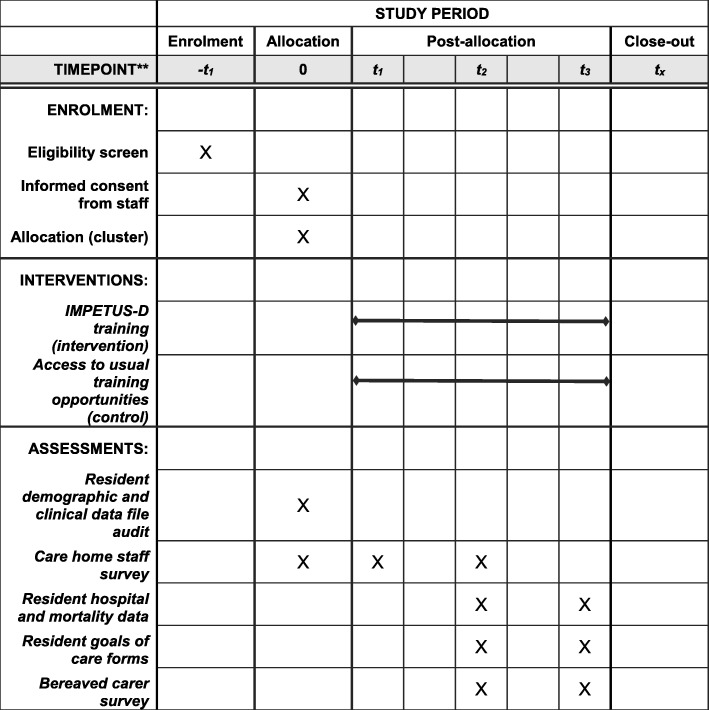
Schedule of enrolment, interventions, and assessments for the IMPETUS-D study. t_1_: 2-month training period**.** t_2_: 6-months follow up. t_3_: 12-months follow up

Figure 1 outlines the Schedule of enrolment, interventions, and assessments for the IMPETUS-D study.

### Control group

Staff from RACFs in the control group will not receive the IMPETUS-D training but will be able to receive usual training opportunities which includes training that may be provided by the facility or health care professionals visiting the facility, or may involve no training during the trial timeframe.

### Outcome measures

#### Primary outcome

The primary composite outcome measure is: 20% reduction in proportion of hospital transfers or deaths in hospital over 6 months in the intervention group (IMPETUS-D) compared to the control group.

#### Secondary outcomes

Secondary outcomes are:

- Proportion of hospital transfers or deaths in hospital over 12 months
- Proportion of hospital transfers over 6 and 12 months
- Proportion of deaths in RACF over 6 and 12 months
- Uptake of Goals of Care plans over 6 and 12 months
- Change in knowledge and attitudes about palliative care for people with advanced dementia following training and at 6 months follow up

### Process evaluation

- Discrepancies between the implementation plan and its operationalization
- Intervention activities that took place and who conducted the intervention activities
- Uptake of the training program: proportion of PCAs and nursing staff at the intervention sites who completed the training - number of logins, time spent on each module, number of modules completed

### Qualitative research measures

- Improved staff and family perception of and satisfaction with care
- Acceptability of the intervention
- Barriers to uptake of the IMPETUS-D training program
- Enablers to uptake of the IMPETUS-D training program

### Data collection

Table 2 summarises the data collection methods that will be undertaken to collect primary and secondary outcome measures and baseline resident demographic and clinical data.

**Table 2:** Outcome measures, data collection methods and time points by participant group

Participants	Time points	Methods	Variables/measures
Residents	Baseline	Review of residents’ files	Demographic and clinical data of residents, including: Age, sex, time living in RACF, chronic comorbidities, dementia diagnosis and type of dementia; current level of care and function, ACP or GOC plan in place, hospitalisations/infections/falls in previous 3 months
	6 and 12-months follow-up	Review of residents’ files	Hospital transfers and primary reason for transfer; Death and place of death; GOC plan in place; GOC plan adhered to
RACF staff	Baseline, after training period and 6-months follow-up	Validated survey qPAD	Knowledge and attitudes about palliative and EOL care for people with dementia

*Abbreviations*: *ACP* advance care plans, *EOL* end-of-life, *GOC* goals of care, *qPAD* questionnaire palliative care for advanced dementia, *RACF* residential aged care facility

Resident demographic characteristics and clinical information, including whether an advance care plan (ACP) or GOC plan is in place, will be collected from the RACF resident files. During the follow-up period, any changes in uptake of ACP or GOC plans and whether they were adhered to in relation to transfers to hospital and place of death will be extracted from RACF files.

RACF staff’s knowledge and attitudes about palliative and EOL care for people with dementia will be assessed at baseline, immediately after the training period and at 6-months follow-up using the Questionnaire on Palliative Care for Advanced Dementia (qPAD) [19]. The qPAD is a validated 2-part instrument consisting of 23-item knowledge test and 12-item attitude scale.

#### Primary outcome data

Unplanned transfers to hospital, including the primary reason for transfer, and deaths of residents, date of death and place of death (RACF, hospital, other) will be extracted from the RACF files throughout the 6- and 12-month follow up period.

### Participants and recruitment

Routinely collected data from the files of residents living permanently in the RACFs will be used in the study. Residents will not be required to undertake any additional assessments or tests and they will not be receiving any additional treatment or intervention. Residents and their family will be provided with plain language information about the study via the RACF noticeboards and newsletters.

The Project Coordinators will visit the RACFs to explain the study to staff and encourage participation. All staff will be invited to participate via email, face-to-face at staff meetings, and via notices in staff rooms. Plain language statements will be provided in hard copy and or electronically via email to all potential participants and consent obtained from those willing to participate. Participation by RACF staff is voluntary.

### Process evaluation

A mixed methods process evaluation will be conducted alongside the cluster RCT. As described in the Medical Research Council’s evaluation framework, the process evaluation will aim to ‘explain discrepancies between expected and observed outcomes, to understand how context influences outcomes, and to provide insights to aid implementation’ [17].

The aims of the process evaluation are:

(i) to explore the impact of contextual factors on the delivery and effectiveness of IMPETUS-D, including barriers and facilitators;
(ii) to assess how the intervention was delivered and achieved, capturing implementation fidelity and dose; and
(iii) to explore participants’ experience and responses to the intervention, including reach, acceptability, satisfaction and knowledge acquisition.

Table 3 summarises the process evaluation data collection activities by CFIR domain [16]. Qualitative and quantitative data collection methods include purposive sampling for semi-structured individual and group interviews as well as participant surveys during the pre- and post-intervention phase.

**Table 3:** Process evaluation research questions and data collection methods by Consolidated Framework for Implementation Research (CFIR) domain

Research question	CFIR domain	Data sources and timeline
What contextual factors (barriers and facilitators) influence the delivery and impact of the intervention?	Outer setting • External policy and incentives	Pre-intervention/baseline Website search and discussions with Project Advisory Group experts to identify palliative care external policy and incentives, accreditation and standards.
	Inner setting • Structural characteristics • Learning climate • Networks and communications • Readiness for implementation	Pre-intervention/baseline Survey general managers to collect data on structural characteristics, learning climate and readiness for implementation. Project Coordinators to collect information on additional structural characteristics. Group/individual interviews with senior staff to explore current practice, networks and communications, and learning climate. During early stages of training period Project Coordinators to collect information on barriers and facilitators to implementing intervention at each intervention care home.
	Characteristics of individuals • Knowledge and beliefs	Pre-intervention/baseline Survey (qPAD) care staff to assess knowledge and beliefs on advanced dementia palliative care; barriers and enablers to providing quality advanced dementia care.
To what degree is the intervention implemented as planned? Assess recruitment, reach, dose (quantity), fidelity (quality).	Process • Planning • Engaging • Executing	During 2-month training period Participation and module completion rates via the LMS. Survey embedded in modules to assess participant satisfaction and module feedback. Project coordinator documentation of implementation activities undertaken, local challenges and strategies used to overcome issues. Shortly after the training period Semi-structured interviews with Project coordinators to explore their experience of implementation. Semi-structured group/individual interviews with staff from the intervention group to assess their experience of implementation.
How does the delivered intervention produce change? Assess participants’ responses to and interactions with the intervention, mediators and unexpected pathways and consequences.	Characteristics of individuals • Knowledge and beliefs • Individual stage of change	6-months follow up Semi-structured group/individual interviews with staff from the intervention group about their experience with and impact of the intervention. Repeat survey (qPAD) of care staff to assess knowledge and beliefs on advanced dementia palliative care.

*Abbreviations*: *LMS* learning management system, *qPAD* questionnaire of palliative care in advanced dementia

### Data management

A dedicated study database has been developed using the Research Electronic Data Capture (REDCap) electronic data capture tools hosted at the University of Melbourne [20]. REDCap is a secure, web-based application designed to support data capture for research studies, providing 1) an intuitive interface for validated data entry; 2) audit trails for tracking data manipulation and export procedures; 3) automated export procedures for seamless data downloads to common statistical packages; and 4) procedures for importing data from external sources. Paper documentation will be securely stored in a locked filing cabinet. All records will be kept for a minimum of 5 years post study completion. Only listed research staff working on the project will have access to these files.

### Power calculation

In this study 24 facilities (12 intervention sites and 12 control sites) will be recruited. The power analyses were based on the following assumptions: proportion of composite events of 0.65 and 0.85 over 6 months of follow-up in the intervention and control groups respectively, two-sided significance level of 0.05 (alpha), 12 clusters (facilities) in each study arm, 20–25 residents with dementia in each cluster, an Intra-Cluster Correlation (ICC) of 0.05. With these assumptions, the minimum power to observe a difference of proportion by 0.20 between the intervention and control group is 94%. If the number of residents with dementia per cluster reduces to 15 only, the power to observe this difference reduces to 0.93%. If the cluster size in each group is reduced to 10 and the number of residents in each cluster is only 15, then the study would have a power of 88% to observe a difference in proportion by 0.20.

The assumptions on the composite of events for this study were based on the information reported in the study by RS Martin (2017) [21].

### Statistical methods

All analyses will be conducted on an intention-to-treat basis. The basic statistics will be presented by number (%), mean (SD), median (Q1, Q3), mean (95% CI) or median (95% CI of median), as appropriate. For analyses of primary and secondary outcomes, the fundamental principle of analysing data from cluster randomised trials will be followed, where appropriate adjustments for cluster level variations will be accounted for to ensure robust inferences.

To compare the proportion of composite events for the primary outcome and the proportions for individual components of the primary outcome (secondary outcomes), logistic regression models will be used with standard errors weighted by the cluster effects. The uptake of GOC is a binary measure at the individual resident level, and will be analysed using logistic regression. Odds ratios and 95% confidence interval will be presented, along with the *p* value, for comparison between intervention and control groups. The change in RACF staff’s knowledge and attitudes about palliative and EOL dementia care immediately following the training period and at 6-months from baseline will be measured using the qPAD. The median change in qPAD score immediately following training and at 6-months follow-up will be compared between the study groups using quintile regression model (adjusted for cluster effects). All statistical tests will be two-sided with a significance level of 0.05.

Additional exploratory analyses will be conducted to evaluate the primary and secondary outcomes of the study, adjusting for potential confounders such as age, sex and comorbidities.

## Discussion

With the ageing population, the number of people with dementia is expected to increase, and so too will the need for high quality advanced dementia care delivered in RACF settings. This cluster RCT will evaluate the effectiveness of an innovative screen-based simulation training program on caring for people with advanced dementia, aimed at staff working in the residential care setting. This large multisite trial will provide robust evidence about the impact of the intervention.

In this study we assess a new and innovative screen-based simulation training program. The training program is designed to address the current status of the RACF workforce in Australia, including high staff turnover, varying skill sets and literacy levels. The training resource has been developed with input from the end-users and subject matter, education and simulation experts. Furthermore, being screen-based and accessible online will allow the often time poor residential care workers, greater convenience and opportunity to participate.

The modules include narratives derived from experts’ experiences and in consultation with residential care staff. The modules aim to achieve functional task alignment (the simulator does what is required to meet the learning objective) and learner engagement (the extent to which the learner participates or ‘buys-in’ to in the activity to meet the learning objective) – two concepts considered more valuable than ‘fidelity’ [22]. The narratives went someway to addressing these concepts through development with those intimately acquainted with this work from provider and user perspectives, by shooting the videos and selected still images in local facilities and seeing and hearing the voices of actual care staff in narratives.

The generalisability of findings from studies involving multiple RACFs is always a challenge. However, various aspects of heterogeneity of the RACFs in different Australian cities have been considered in this cluster randomised study, and block design in the randomisation has been used. The study is adequately powered under different possibilities, allowing us to draw robust inference.

The process evaluation has been designed to explore the degree to which IMPETUS-D is implemented as intended, the impact of contextual factors on intervention delivery, and the participants’ response to and interactions with the intervention. A mixed methods approach has been incorporated in this process evaluation – using qualitative research methods (including individual and group interviews), as well as quantitative methods (including self-report surveys, automated online data collection). Where effectiveness varies between care homes, the process evaluation will assist in assessing the contextual factors and implementation processes associated with effectiveness and differences in outcomes. The process evaluation will provide valuable information that helps explain and interpret the results of the cluster RCT.

Finally, the demand for high quality EOL dementia care in residential care homes is growing. It is imperative the care workers have the skills and knowledge to provide high quality palliative care. If the IMPETUS-D training program is shown to be effective, it will be made available to care homes nationally via a dementia training website.

## Supplementary information

**Additional file 1:** Summary of the implementation strategies that will be conducted as part of the study.

## Data Availability

Data will be available after analyses is finalised and report / publication has been submitted and approved. Unidentifiable individual participant data and related data dictionaries will be available. Access is subject to approval by the Principal Investigator.

## Abbreviations

ACP: Advance care plans
CFIR: Consolidated framework for implementation research
EOL: End-of-life
GOC: Goals of care
IMPETUS-D: IMproving Palliative care Education and Training Using Simulation in Dementia
LMS: Learning management system
qPAD: Questionnaire palliative care for advanced dementia
RACF: Residential aged care facility
RCT: Randomised controlled trial
