# Supplementary material for: A screen-based simulation training program to improve palliative care of people with advanced dementia living in residential aged care facilities and reduce hospital transfers: study protocol for the IMproving Palliative care Education and Training Using Simulation in Dementia (IMPETUS-D) cluster randomised controlled trial
Source: BMC Palliat Care. 2019 Oct 23;18:86. doi: 10.1186/s12904-019-0474-x (PMC6813113; doi:10.1186/s12904-019-0474-x)
Supplement: Supplementary file 1 — Additional file 1: Summary of the implementation strategies that will be conducted as part of the study. [file 12904_2019_474_MOESM1_ESM.docx]

**Additional file 1: Specifications of the implementation strategies, as proposed by Proctor et al 2013 (ERIC discrete implementation strategy compilation, Powell B et al 2015)**

| **Name of ERIC strategy** | **Actors** | **Actions** | **Target(s) of the action** | **Temporality** | **Dose** | **Implementation outcome affected** |
| --- | --- | --- | --- | --- | --- | --- |
| Obtain formal commitments | Principle researcher | Obtain written commitment from key partner | Care home Executive | At commencement of project | Once off | Uptake of the intervention |
| Mandate change | Care home executive/ leaders | Leadership send email to care home staff stating the priority of the innovation and their determination to have it implemented | Care home staff | 2 months prior to the innovation training period | Once off | Uptake of the intervention |
| Recruit local Project coordinators | Care home executive | Recruit Project coordinators who will have dedicated time to assist in implementation at the local level | Project coordinators | 3-4 months prior to the innovation training period | Once off | Fidelity to the implementation plan and study protocol |
| Conduct Project coordinator training with manual | Research team | Hold face to face training day | Project coordinators | 2-3 months prior to the innovation training period | Once off | Fidelity to the implementation plan and study protocol |
| Assess for readiness and identify barriers and facilitators | General mangers  Project coordinators  Research team | Care home visits, interviews and survey to assess readiness to implement | General managers, Senior care home staff | Baseline and mid- training period | Once off survey; and qualitative interviews | Feasibility and fidelity |
| Conduct educational outreach visits | Project coordinators | Care home visits to explain benefits of the intervention | Care home staff | During the 2-month training phase | Presentations delivered to groups of staff | Uptake of the intervention; participation in the program |
| Marketing | Project coordinators | Care home visits, flyers and emails or texts to promote intervention uptake | Care home staff | During the 2-month training phase | 1-2 visits to each care homes; once off flyers, emails and texts | Uptake of the intervention; participation in the program |
| Remind target group | Project coordinators  Care home managers | Reminders sent via email or text, attend staff meetings at low uptake sites | Care home staff | During the 2-month training phase | Fortnightly for 8 weeks; or weekly at sites with low uptake | Uptake of the intervention; participation in the program |
| Audit and provide feedback | Project coordinators  Research team | Collect and summarise training participation rates | Care home staff, including general managers; Care home executive | During the 2-month training phase | Weekly at low uptake sites; otherwise fortnightly | Uptake of the intervention; participation in the program |
| Tailor strategies | Project coordinators  Research team | Tailor the implementation strategies to address barriers and leverage facilitators | Care home staff | During the 2-month training period | Fortnightly or as required | Uptake of the intervention; participation in the program |
